# College and Hospital Notes

**Published:** 1909-05

**Authors:** 


### COLLEGE AND HOSPITAL NOTES

The sixty-third annual commencement of the Medical Department of the University of Buffalo, will be held at the Teck Theater, Friday, May 28, 1909 at 11 A. M. The program is not yet out but the usual ceremonies, doubtless, will be observed. The Alumni Association will begin its annual conclave Friday evening, May 25, with a reception, addresses, election of officers, and a collation. During Wednesday and Thursday, clinics, luncheons, and other entertainments will take place.

